# Risk factors for surgical site infection following posterior internal fixation of thoracolumbar fractures

**DOI:** 10.3389/fsurg.2026.1741838

**Published:** 2026-06-03

**Authors:** Pengxi He, Lei Ren, Shenshen Hao, Chengjin Zhao, Zhibin Liu, Changhong Li, Yangyang Feng, Yong Feng, Yuhu Zhou, Nannan Li

**Affiliations:** 1 Department of Orthopedics, Affiliated Hospital of Yan’an University, Yan’an, Shaanxi Province, China; 2 First Orthopedic Ward, Yan'an People's Hospital, Yan'an, Shaanxi Province, China; 3 Department of Spine, Affiliated Hospital of Qingdao University, Qingdao, Shandong Province, China; 4 Department of Spine and Bone Oncology, General Hospital of Pingmei Shenma Medical Group, Pingdingshan, Henan Province, China

**Keywords:** albumin, posterior internal fixation, preoperative bleeding, risk factors, surgical site infection, thoracolumbar fractures

## Abstract

**Background:**

Surgical site infection (SSI) following posterior internal fixation of thoracolumbar fractures constitutes a severe complication that adversely impacts patient recovery. Despite its clinical significance, there remains a paucity of research investigating the risk factors associated with SSI. Therefore, this study aimed to explore these risk factors and propose potential therapeutic strategies.

**Methods:**

A retrospective analysis was conducted on 157 patients who underwent posterior internal fixation for thoracolumbar fractures at the Department of Orthopedics, Affiliated Hospital of Yan’an University, between February 2017 and October 2021. Patients were stratified into an infection group (*n* = 12) and a non-infection group (*n* = 145). Preoperative baseline data included age, gender, body mass index, total protein (TP), albumin (ALB), hemoglobin (HB), red blood cell count (RBC), white blood cell count (WBC), total lymphocyte count (TLC), platelet count (PLT), and the presence of diabetes or hypertension. Operative data encompassed preoperative preparation time, length of the surgical incision, number of internal fixation segments, operation duration (≥3 h vs. <3 h), occurrence of allogeneic blood transfusion, intraoperative blood loss (≥400 mL vs. <400 mL), and type of drainage tube (negative-pressure vs. normal). Postoperative management-related data included dressing changes at the surgical incision within 24 h following the operation, indwelling time of the drainage tube (≥3 days vs. <3 days), drainage volume upon removal of the drainage tube (≥50 mL vs. <50 mL), reoperation requirement, and postoperative levels of TP, ALB, HB, RBC, WBC, TLC, and PLT. Eligible data were subjected to univariate analysis, followed by multivariate logistic regression for variables with significant univariate associations.

**Results:**

Univariate analysis revealed significant differences between groups in intraoperative blood loss (≥400 mL), preoperative ALB (<30 g/L), preoperative RBC, postoperative HB, postoperative RBC, surgical incision length, and number of internal fixation segments between groups (all *P* < 0.05). Multivariate logistic regression analysis identified preoperative hypoalbuminemia (<30 g/L) [odds ratio (OR) = 0.851, *P* = 0.028] and intraoperative blood loss (≥400 mL) (OR=7.477, *P* = 0.005) as independent risk factors for SSI.

**Conclusion:**

Preoperative hypoalbuminemia (<30 g/L) and intraoperative blood loss (≥400 mL) are associated with an elevated risk of SSI following posterior internal fixation of thoracolumbar fractures. Implementing effective perioperative management strategies is crucial to mitigate the incidence of SSI.

## 1 Background

Posterior internal fixation is one of the most prevalent surgical approaches for thoracolumbar fractures. Thoracolumbar fractures account for approximately 50% of all spinal fractures, with high-energy injuries such as traffic accidents and falls from heights being the primary causes (34). Posterior internal fixation has a clinical application rate of more than 80% for this type of fracture due to its advantages of clear surgical field, firm fixation, and rapid postoperative recovery. Such fractures predominantly affect active young and middle-aged adults, with vertebral burst fractures being the primary fracture type. Clinical manifestations are closely related to the degree of nerve compression. However, the surgical trauma and spinal stability damage associated with this procedure also increase the risk of postoperative complications, among which surgical site infection (SSI) is the most common and harmful one (1, 2). For patients with SSI, decompression combined with internal fixation serves as the cornerstone of treatment, as this mode of treatment enables the rapid and effective removal of nerve-compressing soft tissues and bone fragments, thereby promoting the recovery of compressed nerves. However, postoperative complications of spinal internal fixation, most notably SSI (3, 4), not only lead to prolonged hospital stay (an average of 10–14 days longer than non-SSI patients) and a two to threefold increase in medical costs (35) but also easily cause serious sequelae, such as spinal fusion failure, chronic low back pain, permanent nerve function damage, and increased risk of reoperation and in-hospital mortality (30). According to a recent multicenter study (9), the incidence of SSI following posterior internal fixation for thoracolumbar fractures ranges from 2.3% to 15.6%, which is significantly higher than that of other spinal surgeries due to severe trauma and poor soft tissue condition (5, 6, 33).

For patients with SSI after spinal surgery, clinical recovery is typically attainable via pharmacotherapy and debridement. Nevertheless, some patients who undergo posterior internal fixation or fusion face refractory infections due to extensive infection foci and complex clinical conditions, resulting in suboptimal outcomes with early conservative management. When fixed segments fail to meet fusion criteria, implant removal is indicated (accounting for the potential formation of bacterial biofilms on the implant surface), which once again compromises spinal stability. These patients require elective revision fixation. However, the uncertainty surrounding treatment outcomes imposes substantial economic burdens and severe physical trauma on both patients and their families. Thus, the prevention of SSI is of paramount clinical significance.

Against this backdrop, the present study aimed to investigate the risk factors for SSI following thoracolumbar fracture surgery, proposed evidence-based treatment strategies, summarized and analyzed targeted preventive measures, and provided a reference for reducing the incidence of SSI.

## 2 Methods

### 2.1 Study design

This study was a single-center, retrospective case analysis, approved by the Ethics Committee of the Affiliated Hospital of Yan’an University (Approval No. IIT-R-20250196). All study procedures were conducted in strict compliance with the ethical principles stipulated in the Declaration of Helsinki. Clinical data of patients with thoracic, lumbar, or thoracolumbar fractures who underwent posterior internal fixation at the Department of Orthopedics, Affiliated Hospital of Yan’an University, between February 2017 and October 2021, were collected and analyzed. The inclusion criteria were as follows: (a) surgical incision classified as Class I; (b) fractured segment involving the thoracolumbar spine; (c) posterior internal fixation performed; (d) surgery performed by physicians with the title of associate chief physician or higher, with at least 10 years of clinical experience in orthopedics; and (e) complete medical records available. The exclusion criteria were as follows: (a) history of primary spinal infectious diseases; (b) history of immune system disorders; (c) history of malignant tumors; (d) age less than 18 years (7); and (e) transfer to the intensive care unit (ICU) during diagnosis and treatment.

**Figure 1:**
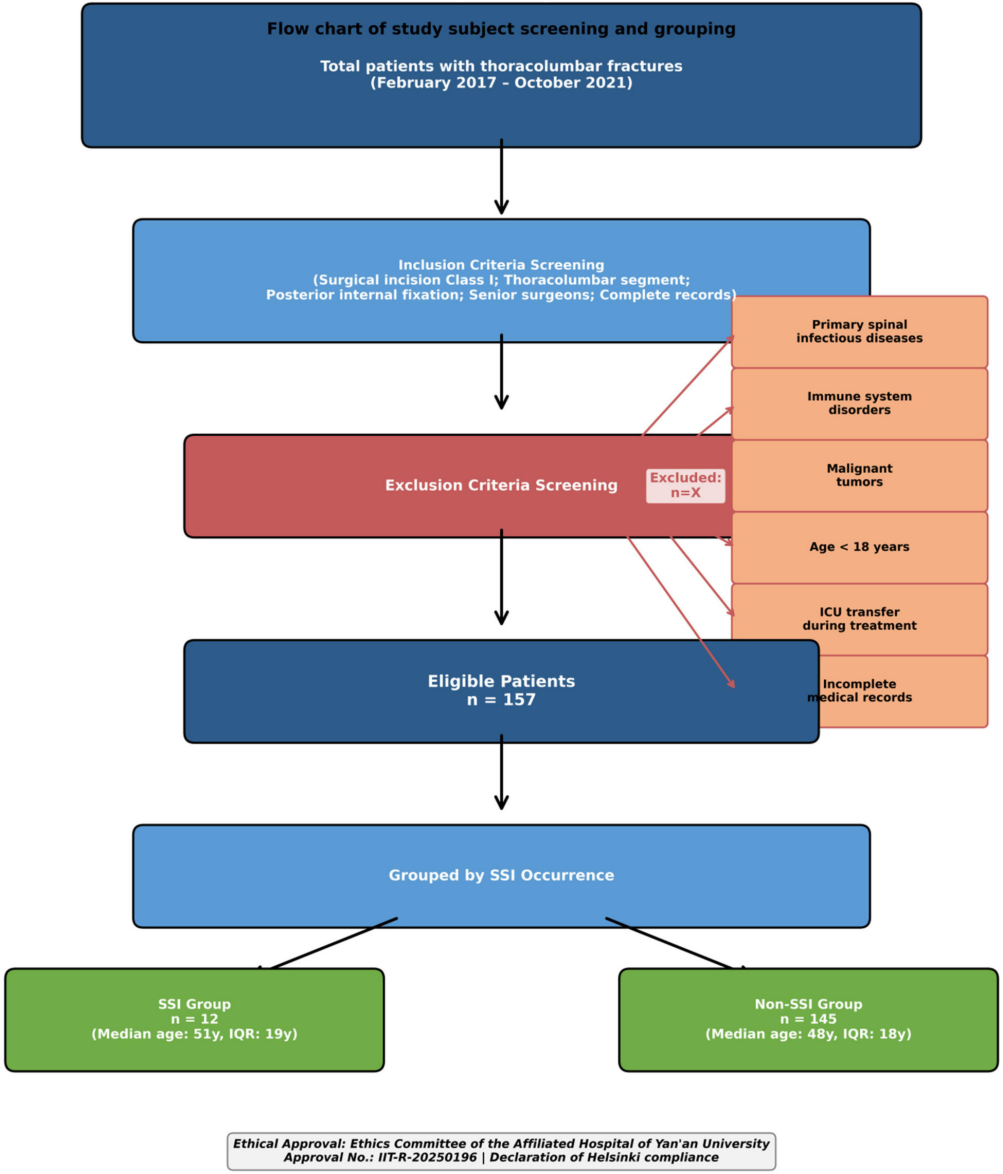
Flow chart of study subject screening and grouping. The inclusion and exclusion criteria were applied strictly in accordance with the ethical approval granted by the Ethics Committee of the Affiliated Hospital of Yan'an University (Approval No. IIT-R-20250196).

A total of 157 eligible patients were included, consisting of 111 men and 46 women. Patients were stratified into non-SSI and SSI groups. For the non-SSI group, the median age (M) was 48 years with a quartile range (QR) of 18 years; for the SSI group, the median age was 51 years with a QR of 19 years (Figure 1).

### 2.2 Diagnostic criteria for SSI

According to the specifications of the National Healthcare Safety Network—a component of the US Centers for Disease Control and Prevention—the diagnosis of SSI requires meeting any one of the following criteria (8): (a) presence of purulent exudate in the superficial and deep layers of the incision; (b) pathogenic microorganisms cultured from incision tissue or body fluids; and (c) evidence of infection or signs and symptoms of inflammatory response identified via direct physical examination, imaging studies, reoperation, pathological examination, or other modalities.

### 2.3 Research methods

All patients underwent surgery under general anesthesia in the prone position. Following comprehensive preoperative preparation and successful anesthesia induction, the surgical procedure was initiated. Preoperative imaging findings were utilized to localize the injured vertebra(e). The anatomical location of the injured vertebra(e) was confirmed using either surface anatomical landmarks or C-arm fluoroscopy. A longitudinal incision of appropriate length was made over the fracture site. Sequential dissection was performed through the skin, subcutaneous tissue, and deep fascia. During the standard surgical procedure, the injured vertebra(e) and the pedicle screw insertion sites of the adjacent superior and inferior vertebrae were exposed. Pedicle screws were inserted sequentially into the injured vertebra(e) (either unilaterally or bilaterally) and into the bilateral pedicles of the adjacent vertebrae at varying trajectories. Based on preoperative imaging results and the patient’s clinical manifestations, decompressive laminectomy and removal of intraspinal bone fragments were performed as indicated to alleviate spinal cord and nerve root compression. Appropriate connecting rods were then placed and distracted to facilitate fracture reduction and spinal stabilization. The position of the internal fixation constructs was verified via C-arm fluoroscopy. The wound was irrigated with 3,000–5,000 mL of normal saline combined with antibiotics during the operation. Hemostasis was achieved using gelatin sponge at the bleeding site. Prophylactic antibiotics were administered intravenously 30 min before the operation and continued for 24–48 h after the operation. All surgical operations were standardized by the same team of senior orthopedic surgeons to ensure the consistency of surgical procedures. After thorough wound irrigation, a drainage tube was placed and the incision was closed in layers.

### 2.4 Research indicators

The observational indices of this study were categorized into preoperative baseline data, operative-related variables, and postoperative management-related data. Preoperative baseline variables included age, gender, body mass index, total protein (TP), albumin (ALB), hemoglobin (HB), red blood cell count (RBC), white blood cell count (WBC), total lymphocyte count (TLC), platelet count (PLT), and the presence of diabetes or hypertension. Operative-related data encompassed preoperative preparation time, length of the surgical incision, number of internal fixation segments, operation duration (≥3 h vs. <3 h), occurrence of allogeneic blood transfusion, intraoperative blood loss (≥400 mL vs. <400 mL), and type of drainage tube (negative-pressure vs. normal). *Postoperative* management-related *data* included dressing changes at the surgical incision within 24 h after operation, indwelling time of the drainage tube (≥3 days vs. <3 days), drainage volume upon removal of the drainage tube (≥50 mL vs. <50 mL), reoperation requirement, and postoperative levels of TP, ALB, HB, RBC, WBC, TLC, and PLT.

### 2.5 Statistical methods

Continuous variables were first tested for normality using the Shapiro–Wilk test (*P* > 0.05 indicated normal distribution) and then for homogeneity of variance using the Levene test (*P* > 0.05 indicated homogeneous variance). Normally distributed variables with homogeneous variance were expressed as mean ± standard deviation ($\bar{x}$ ± s) and compared between groups using the independent-samples t-test; normally distributed variables with heterogeneous variance were compared using Welch’s *t*-test. Non-normally distributed variables were expressed as median and interquartile range [M (P25, P75)] and compared between groups using the Mann–Whitney U test.

Count data were expressed as *n* (%) and analyzed using the chi-square test. When the theoretical frequency T < 5 or the total sample size *n* < 40, Fisher’s exact probability test was uniformly used for comparison, with the results were marked by an asterisk (*) in tables.

Variables with *P* < 0.05 in univariate analysis were included in the multivariate logistic regression analysis, and the forward stepwise method was used for variable screening with the test level *α* = 0.05. Odds ratios (ORs) and 95% confidence intervals (95% CI) were calculated to evaluate the correlation strength between variables and SSI.

For continuous variables with significant differences, stratified analysis was performed according to clinically common critical values and univariate results: intraoperative blood loss was divided into <200 mL, 200–400 mL, and ≥400 mL groups; preoperative ALB was divided into <30 g/L, 30–40 g/L, and ≥40 g/L groups to explore the threshold effect and dose–response relationship.

**Figure 2:**
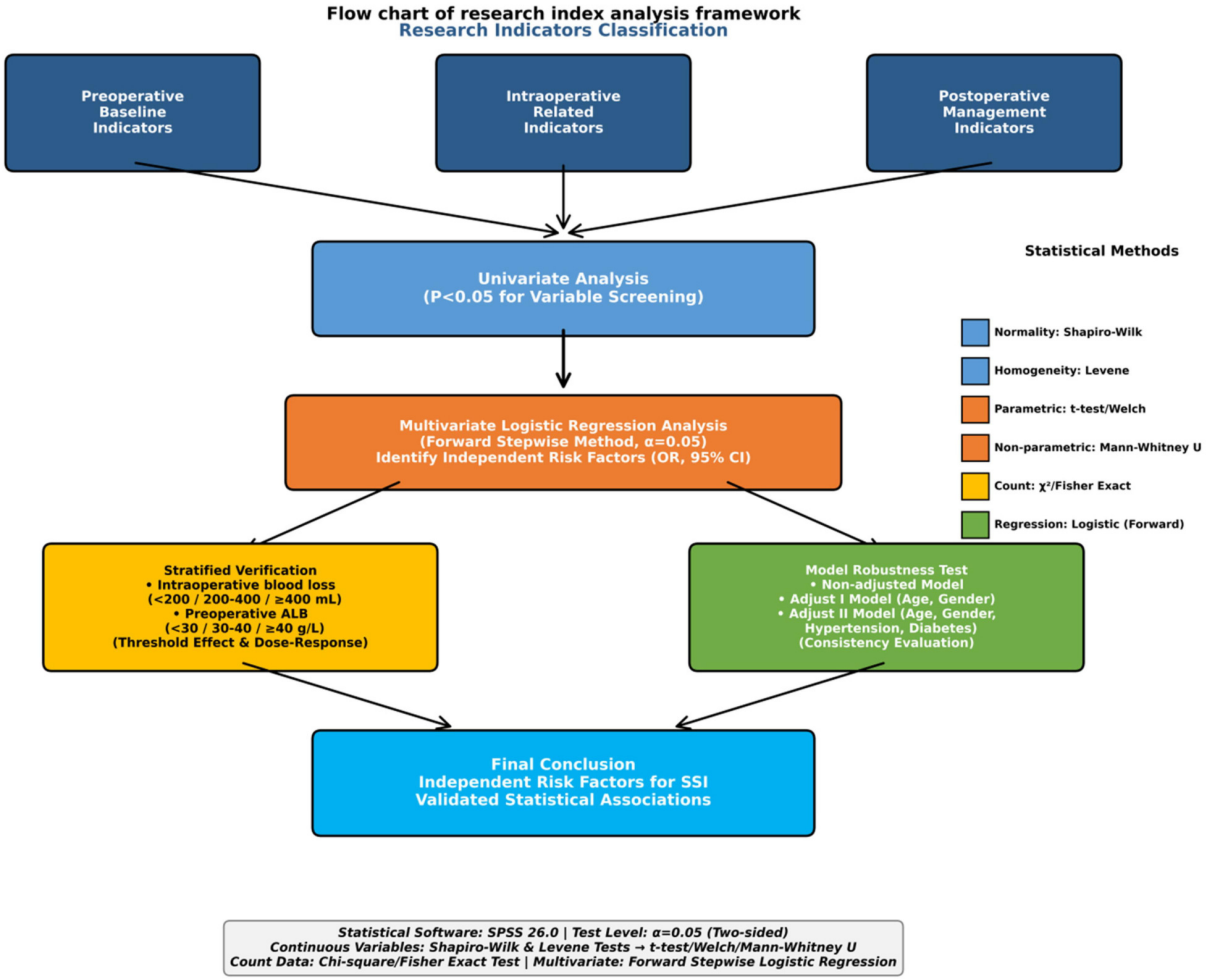
Flow chart of research index analysis framework. All statistical analyses were performed using SPSS 26.0, and the test level was *α* = 0.05 (two-sided test).

Model robustness was verified by three models: non-adjusted model (no correction), Adjust I model (corrected for age and gender) and Adjust II model (corrected for age, gender, hypertension, and diabetes mellitus). The consistency of results across the different models was used to evaluate the independence of risk factors (Figure 2).

## 3 Results

### 3.1 Participant data analysis

A total of 157 patients were divided into an SSI group (*n* = 12) and a non-SSI group (*n* = 145). All patients underwent posterior internal fixation, and those with SSI underwent debridement and were discharged.

Univariate analysis of the predefined variables (emphasizing *a priori* study design to avoid bias) revealed statistically significant differences (*P* < 0.05) in the following indices: intraoperative blood loss (≥400 mL), preoperative ALB (≤30 g/L), preoperative RBC, postoperative HB, postoperative RBC, surgical incision length (cm), and number of internal fixation segments (Table 1). Univariate analysis results showed that there were statistically significant differences in seven indicators between the SSI group and the non-SSI group (all *P* < 0.05), including intraoperative blood loss ≥400 mL, preoperative ALB < 30 g/L, preoperative RBC, postoperative HB, postoperative RBC, surgical incision length, and number of internal fixation segments. The proportion of patients with intraoperative blood loss ≥400 mL in the SSI group was 75.0%, which was 3.23 times higher than that in the non-SSI group (23.2%), suggesting a strong correlation between massive intraoperative bleeding and SSI. The median preoperative ALB in the SSI group (36.60 g/L) was lower than that in the non-SSI group (39.30 g/L), indicating that low preoperative albumin levels may increase the risk of SSI. In addition, no statistically significant differences were found in age, gender, diabetes, hypertension, and other indicators between the two groups (all *P* > 0.05), which excluded their potential as single-factor risk factors for SSI. After adjusting for potential confounding factors, multivariate logistic regression analysis identified two independent risk factors for SSI after posterior internal fixation for thoracolumbar fractures: preoperative hypoalbuminemia (≤30 g/L) (OR = 0.851, 95% CI: 0.737–0.983, *P* = 0.028) and intraoperative blood loss ≥400 mL (OR = 7.477, 95% CI: 1.812–30.856, *P* = 0.005) (Table 2). For every 1 g/L increase in preoperative ALB, the risk of SSI decreased by 14.9%, indicating its protective effect; patients with intraoperative blood loss ≥400 mL had a 7.477-fold higher risk of SSI than those with blood loss <400 mL, suggesting that it is a strong independent risk factor.

**Table 1:** Univariate analysis of SSI group and non-SSI group.

Project	SSI (*n* = 12)	Non-SSI (*n* = 145)	t/*χ*^2^/Z value	*P*-value
Age (years)	51.00 (19.00)	48.00 (18.00)	−0.331	0.741
Gender				
Male	8 (66.7%)	103 (71.1%)	0.000	1.000
Female	4 (33.3%)	42 (29.0%)		
Diabetes				
Yes	0 (0.0%)	4 (2.8%)	-	1.000*
No	12 (100.0%)	141 (97.2%)		
Hypertension				
Yes	2 (16.7%)	7 (4.8%)	-	0.143*
No	10 (83.3%)	138 (95.2%)		
Surgical succession				
Yes	8 (66.7%)	80 (55.2%)	0.594	0.441
No	4 (33.3%)	65 (44.8%)		
Operation time (≥3 h)				
Yes	7 (58.3%)	57 (39.3%)	0.967	0.326
No	5 (41.7%)	88 (60.7%)		
Allogeneic blood transfusion				
Yes	4 (33.3%)	19 (13.1%)	2.190	0.139
No	8 (66.7%)	126 (86.9%)		
Intraoperative bleeding volume (≥400 mL)				
Yes	9 (75.0%)	33 (23.2%)	12.450	0.000
No	3 (25.0%)	109 (76.8%)		
Drainage tube				
Negative pressure	12 (100.0%)	100 (72.5%)	3.089	0.079
Common	0 (0.0%)	38 (27.5%)		
Drainage tube retention time (≥3 days)				
Yes	9 (90.0%)	83 (59.3%)	2.530	0.112
No	1 (10.0%)	57 (40.7%)		
Drainage volume (≥50 mL) when drain tube is removed				
Yes	1 (12.5%)	21 (14.9%)	0.000	1.000
No	7 (87.5%)	121 (85.1%)		
Change of dressing for surgical incision (24 h)				
Yes	0 (0.0%)	4 (2.8%)	-	1.000*
No	11 (100.0%)	139 (97.2%)		
BMI (kg/m^2^)	22.92 ± 2.74	23.05 ± 3.08	0.111	0.912
Preoperative TP (g/L)	59.47 ± 11.93	64.79 ± 6.94	1.458	0.174
Preoperative ALB (g/L)	36.60 (9.70)	39.30 (5.60)	−1.994	0.046
Preoperative HB (g/L)	121.00 (39.00)	138.00 (30.00)	−1.933	0.053
Preoperative RBC (10^12^/L)	3.83 (1.19)	4.50 (0.81)	−2.441	0.015
Preoperative WBC (10^9^/L)	10.09 (8.33)	8.39 (3.95)	−0.063	0.950
Preoperative PLT (10^9^/L)	156.00 (97.00)	182.00 (69.00)	−0.998	0.318
Preoperatively TLC (10^9^/L)	1.19 (1.43)	1.17 (0.77)	−0.172	0.864
Postoperative TP (g/L)	55.63 ± 9.62	58.11 ± 6.71	0.762	0.467
postoperative ALB (g/L)	31.58 ± 5.89	33.74 ± 3.94	1.088	0.307
Postoperative HB (g/L)	97.00 (23.00)	115.00 (23.00)	−2.244	0.025
Postoperative RBC (10^12^/L)	3.18 (0.69)	3.80 (0.78)	−2.458	0.014
Postoperative WBC (10^9^/L)	8.94 (5.95)	8.71 (3.97)	−0.086	0.932
Postoperative PLT (10^9^/L)	164.00 (82.00)	185.00 (91.00)	−1.776	0.076
Postoperative TLC (10^9^/L)	1.05 (0.62)	1.17 (0.66)	−0.842	0.400
Operative incision length (cm)	16.00 (5.00)	10.00 (2.00)	−2.228	0.026
Preoperative hospital stay (days)	3.00 (5.00)	3.00 (3.00)	−0.335	0.737
Number of internal fixation segments (segment)	4.00 (2.00)	3.00 (1.00)	−2.942	0.003

Normal reference ranges: preoperative ALB (35–51 g/L), preoperative HB (120–160 g/L for males, 110–150 g/L for females), preoperative RBC (4.0–5.5 × 10^¹²^/L for males, 3.5–5.0 × 10^¹²^/L for females). TP, total protein; ALB, albumin; HB, hemoglobin; RBC, red blood cell count; WBC, white blood cell count; PLT, platelet count; TLC, total lymphocyte count; BMI, body mass index.

*Fisher exact probability test.

**Table 2:** Multivariate logistic regression analysis of SSI.

Variable	Wald statistic	OR value	95% Confidence interval	*P*-value
Preoperative ALB (g/L)	4.835	0.851	0.737–0.983	0.028
Intraoperative blood loss (≥400 mL)	7.738	7.477	1.812–30.856	0.005

Wald statistic was used to test the significance of variables in the regression model; OR > 1 indicates a risk factor, OR < 1 indicates a protective factor; 95% CI did not include 1 indicated statistical significance (*P* < 0.05). ALB, albumin.

### 3.2 Continuous variable analysis

Both surgical blood loss and preoperative serum albumin demonstrated significant independent associations with SSI risk when analyzed as continuous variables.

Surgical blood loss: Every 1 mL increase in intraoperative blood loss was significantly associated with increased infection risk across all models. In the fully adjusted model (Adjust II), which controlled for age, gender, hypertension, and diabetes mellitus, the association remained statistically significant (OR = 1.00, 95% CI: 1.00–1.00, *P* = 0.0045). Due to the small unit of measurement (per 1 mL), the OR approximates unity; however, the sustained statistical significance across all adjustment models confirms a dose-dependent positive correlation between blood loss volume and SSI risk.

Preoperative serum albumin: Each 1 g/L increase in preoperative albumin was associated with a 20% reduction in SSI risk in the fully adjusted model (OR = 0.80, 95% CI: 0.68–0.93, *P* = 0.0044). This protective effect remained robust across all adjustment models (non-adjusted: *P* = 0.0080; Adjust I: *P* = 0.0059), demonstrating that higher albumin levels independently protect against postoperative infection regardless of patient demographics or comorbidities (Table 3).

**Table 3:** Multivariable logistic regression analysis of risk factors for SSI.

Exposure	Non-adjusted	Adjust I	Adjust II
Intraoperative blood loss	1.00 (1.00, 1.00) 0.0059	1.00 (1.00, 1.00) 0.0042	1.00 (1.00, 1.00) 0.0045
Preoperative ALB (g/L)			
<30	1	1	1
≥30, <40	0.04 (0.00, 0.52) 0.0138	0.03 (0.00, 0.44) 0.0096	0.03 (0.00, 0.40) 0.0081
≥40	0.02 (0.00, 0.27) 0.0038	0.01 (0.00, 0.21) 0.0025	0.01 (0.00, 0.19) 0.0021
Intraoperative blood loss			
<200	1	1	1
≥200, <400	0.39 (0.03, 4.47) 0.4518	0.42 (0.04, 4.80) 0.4833	0.37 (0.03, 4.46) 0.4363
≥400	6.55 (1.33, 32.26) 0.0210	6.91 (1.38, 34.69) 0.0189	6.85 (1.32, 35.56) 0.0221
Preoperative ALB (g/L)	0.83 (0.72, 0.95) 0.0080	0.81 (0.70, 0.94) 0.0059	0.80 (0.68, 0.93) 0.0044

Non-adjusted model adjust for: None. Adjust I model adjust for: age, sex; Adjust II model adjust for: age, sex, hyper, DM. Data in brackets are 95% confidence interval (95% CI), followed by *p* value; non-adjusted = no correction; Adjust I = corrected for age and gender; Adjust II = corrected for age, gender, hypertension, and diabetes mellitus; ALB, albumin. *p* < 0.05 indicates statistical significance.

### 3.3 Categorical analysis

To further elucidate potential threshold effects and dose–response relationships, both variables were also analyzed categorically.

Preoperative albumin stratification: Using severe hypoalbuminemia (<30 g/L) as the reference group, progressively higher ALB levels showed increasingly stronger protective effects. In the fully adjusted model, patients with ALB levels of 30–40 g/L had a 97% risk reduction (OR = 0.03, 95% CI: 0.00–0.40, *P* = 0.0081), while those with levels ≥40 g/L showed a 99% risk reduction (OR = 0.01, 95% CI: 0.00–0.19, *P* = 0.0021) compared to the reference group. This gradient effect supports a clear dose–response relationship.

Surgical blood loss stratification: Categorical analysis revealed a distinct threshold effect for intraoperative hemorrhage. Compared to patients with minimal blood loss (<200 mL), those with moderate blood loss (200–400 mL) showed no significant difference in infection risk (OR = 0.37, 95% CI: 0.03–4.46, *P* = 0.4363). However, patients with massive hemorrhage (≥400 mL) experienced a nearly sevenfold increased risk of SSI (OR = 6.85, 95% CI: 1.32–35.56, *P* = 0.0221). This suggests that 400 mL represents a critical threshold beyond which infection risk substantially escalates, likely reflecting impaired tissue perfusion, immune dysfunction, and compromised wound healing capacity.

Stratified analysis of continuous variables demonstrated that with the increase of intraoperative blood loss, the risk of SSI increased significantly, and the correlation remained statistically significant in all three adjustment models (*P* < 0.05). For every 1 g/L increase in preoperative ALB, the risk of SSI decreased by about 20% in the fully adjusted model (OR=0.80, *P* = 0.0044), and the protective effect was stable in different models. Stratified analysis of categorical variables revealed obvious threshold and dose–response effects: Using preoperative ALB <30 g/L as the reference group, the SSI risk of 30–40 g/L group decreased by 97% (OR = 0.03), and that of ≥40 g/L group decreased by 99% (OR=0.01). For intraoperative blood loss, there was no significant difference in SSI risk between the <200 mL and 200–400 mL groups (*P* > 0.05), while the risk of the ≥400 mL group increased by 6.85 times (*P* = 0.0221), indicating 400 mL to be the critical threshold of intraoperative blood loss. The consistency of results across non-adjusted, partially adjusted, and fully adjusted models confirmed the robustness of the study results (Table 3).

### 3.4 Model robustness

**Figure 3:**
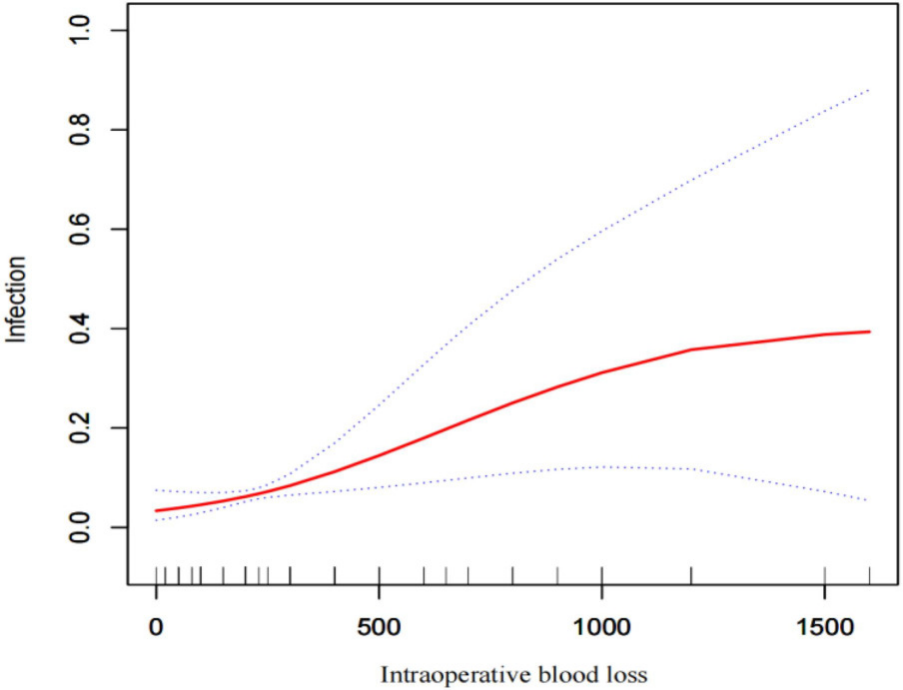
Correlation trend between intraoperative blood loss and SSI occurrence probability. The trend was fitted by smooth curve, and the shaded area represents the 95% confidence interval.

**Figure 4:**
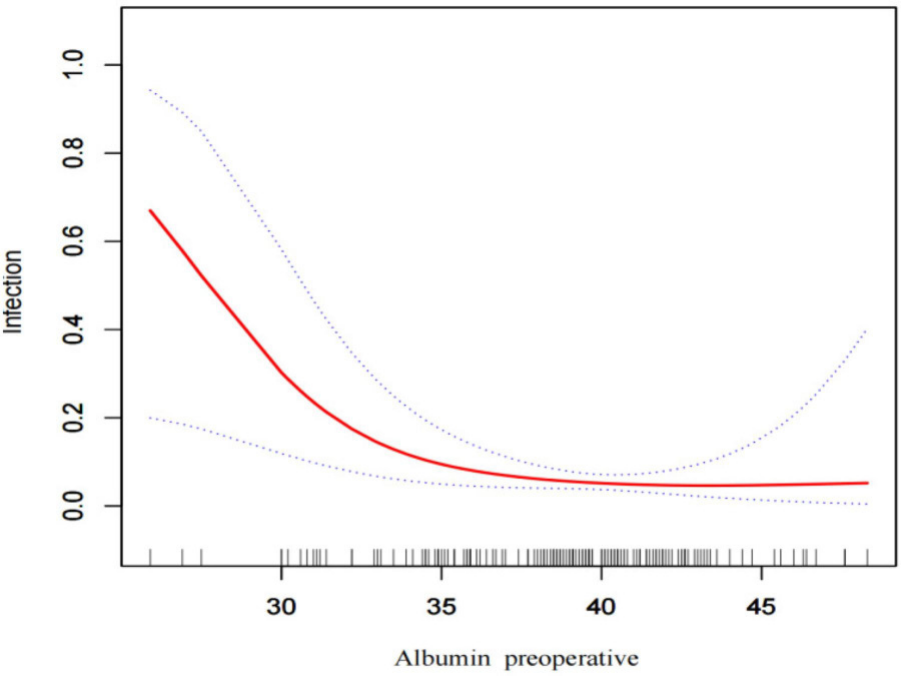
Correlation trend between preoperative ALB level and SSI occurrence probability. The trend was fitted by smooth curve, and the shaded area represents the 95% confidence interval.

The consistency of these associations across non-adjusted, partially adjusted (Adjust I), and fully adjusted (Adjust II) models demonstrates the robustness of these findings and confirms that blood loss and ALB levels are independent risk factors for SSI after posterior instrumentation for thoracolumbar fractures. As shown in Figures 3, 4, there is a significant correlation between the occurrence of infection and the two independent risk factors.

Smooth curve fitting results showed a positive correlation between intraoperative blood loss and SSI occurrence probability: The probability of SSI increased slowly when the blood loss was less than 400 mL and rose sharply after exceeding 400 mL. There was a negative correlation between preoperative ALB level and SSI occurrence probability: The probability of SSI increased significantly when ALB was lower than 30 g/L and decreased gradually with the increase of ALB level. The trend results further verified the threshold effect of the two independent risk factors identified in this study.

## 4 Discussions

### 4.1 Clinical burden of SSI

SSI is a prevalent complication following orthopedic procedures. Existing literature documents that postoperative infections account for 36% of all hospital-acquired infections, with SSIs comprising 1%–3% of these cases (9). Notably, several investigators have emphasized the high clinical burden of spinal surgery-associated SSI, with reported incidence ranging from 0.7% to 12.0% (10), exceeding that of other orthopedic interventions, such as total knee arthroplasty (TKA) or total hip arthroplasty (THA) (11). Thus, the prevention of SSI following spinal surgery is of paramount clinical importance.

SSIs can lead to catastrophic outcomes, including neurological impairment, pseudoarthrosis, sepsis, and mortality. The onset of SSI is associated with prolonged hospital stays, elevated healthcare costs, and measurable psychological distress for both patients and clinicians (12). Reducing the incidence of SSI remains a critical priority for orthopedic surgeons.

### 4.2 Clinical implications of preoperative hypoalbuminemia (<30 g/L)

This study identified preoperative hypoalbuminemia as an independent risk factor for SSI following posterior internal fixation of thoracolumbar fractures. As a principal plasma protein synthesized in the liver (≈12–20 g/day), albumin (ALB) primarily regulates colloid osmotic pressure and serves as a nutritional substrate, with a normal reference range of 35–51 g/L. This threshold enables objective assessment of ALB status.

Perioperative monitoring of ALB is critical for postoperative wound healing. Elderly patients are particularly susceptible to postoperative hypoproteinemia, which contributes to delayed incision healing, prolonged incision exposure, and elevated SSI risk. Prior research has linked lumbar surgery-associated SSI to multiple factors, including age, diabetes mellitus history, preoperative HB levels, American Society of Anesthesiologists classification, fusion segment count, operative duration, ICU transfer, and perioperative allogeneic transfusion (13, 14). Notably, preoperative hypoproteinemia correlates with delayed recovery and suboptimal surgical outcomes (15, 16), highlighting preoperative correction as a key SSI prevention strategy. For thoracolumbar fracture patients, trauma-induced hypermetabolism accelerates ALB catabolism, necessitating prompt intervention to mitigate consumption and correct hypoproteinemia—typically via nutritional optimization or temporary ALB supplementation.

However, conflicting evidence exists. Some investigators report insufficient evidence linking postoperative hypoproteinemia to SSI (17–19), noting no increased SSI risk in spinal surgery patients with postoperative hypoproteinemia compared with normoalbuminemic patients, challenging the premise of preoperative hypoproteinemia as an orthopedic SSI risk factor. Conversely, trauma emergency patients face heightened SSI risk due to complex pathophysiology, immunosuppression, and delayed incision healing (20). Consensus among most orthopedic clinicians, supported by the literature, posits a preoperative hypoproteinemia–SSI association (21–23). Preoperative hypoalbuminemia is also an independent risk factor for SSI and wound complications in general surgery, TKA, THA, and spinal surgery. Postoperative hypoproteinemia affects 70%–80% of patients (24), increasing SSI risk via impaired wound healing, immunosuppression, and persistent inflammation (25).

Daniel Ball et al. (Rush University Medical Center, Department of Plastic Surgery) demonstrated that malnutrition independently predicts SSI after posterior lumbar fusion, correlating with prolonged hospitalization and readmission (26). To clarify hypoalbuminemia’s clinical impact, prospective studies should evaluate the efficacy of preoperative ALB correction on wound healing—an endeavor with the potential to reduce adverse postoperative events.

Based on existing literature and clinical experience, ALB’s nutritional role is well established: Hypoproteinemia causes incision edema, impairs healing, and compromises immunity, reducing incision antimicrobial defenses and increasing infection risk. For patients with preoperative ALB <30 g/L, daily 20 g intravenous ALB is recommended. Clinical observation shows that 10 g/day only provides transient correction (with serum levels returning to baseline by the next day), prolonging treatment duration and increasing costs. These recommendations reflect anecdotal clinical experience, which requires validation via targeted prospective studies.

### 4.3 Clinical implications of intraoperative blood loss ≥ 400 mL

This study identified intraoperative blood loss ≥ 400 mL as an independent risk factor for SSI. HB, a marker of nutritional status, exhibits inverse correlations with blood loss and incision healing—increased intraoperative bleeding and reduced HB levels both impair surgical incision recovery.

Prior research has established a link between surgical blood loss and SSI across surgical specialties (27). In spinal surgery, blood loss exceeding 1 L elevates SSI risk, potentially via mechanisms including reduced effective antibiotic concentrations, tissue hypoperfusion, thermoregulatory instability, and relative immunosuppression. Thus, bleeding volume warrants inclusion into SSI risk stratification. Preoperative planning should prioritize minimizing accidental vascular injury and intraoperative hemorrhage. Moreover, postoperative hidden blood loss has been highlighted as an under-recognized SSI risk factor (28, 29). Intraoperative strategies—including meticulous hemostasis and avoidance of excessive soft tissue traction—mitigate tissue edema, vascular hyperpermeability, and HB depletion, thereby reducing infection susceptibility.

Through correlation analysis of multiple variables, this study confirmed preoperative hypoalbuminemia and intraoperative blood loss ≥ 400 mL as independent SSI risk factors. Given the higher infection burden of spinal surgery relative to other orthopedic procedures, targeted post-surgical surveillance and prophylaxis are imperative. The orthopedic literature reflects inconsistent reporting of SSI risk factors. For example, a study evaluating topical vancomycin for spinal SSI prevention (30) underscored the need for rigorously designed double-blind randomized controlled trials to validate its efficacy. Clinicians should exercise caution when administering vancomycin powder intraoperatively.

In summary, optimizing perioperative management—grounded in iterative clinical experience and interdisciplinary research (31, 32)—is critical for reducing SSI incidence. Concurrently, ongoing education for surgical teams and stakeholders should emphasize infection prevention protocols and the clinical impact of SSI. While complete elimination of infection is unattainable, these measures hold promise for mitigating spinal surgery-associated infection risk.

## 5 Conclusion

This study demonstrates that preoperative hypoalbuminemia and intraoperative blood loss (≥400 mL) are risk factors for SSI following posterior internal fixation of thoracolumbar fractures. For patients with these risk factors, targeted perioperative intervention strategies should be developed and implemented to reduce SSI incidence. Notably, this study has limitations, including a small sample size and low-level evidence. Future research should expand the case database and conduct prospective studies to further validate the correlation between the identified independent risk factors and infection risk.

## Data Availability

The original contributions presented in the study are included in the article/supplementary material; further inquiries can be directed to the corresponding author.
